# Bioinformatic mining for RiPP biosynthetic gene clusters in Bacteroidales reveals possible new subfamily architectures and novel natural products

**DOI:** 10.3389/fmicb.2023.1219272

**Published:** 2023-07-04

**Authors:** Maria Victoria Fernandez-Cantos, Diego Garcia-Morena, Yunhai Yi, Lifeng Liang, Emilio Gómez-Vázquez, Oscar P. Kuipers

**Affiliations:** ^1^Department of Molecular Genetics, Groningen Biomolecular Sciences and Biotechnology Institute, University of Groningen, Groningen, Netherlands; ^2^01Life Institute, Shenzhen, China

**Keywords:** Bacteroidales, microbiota, bacteriocins, RiPP, BAGEL4, rSAM, YcaO

## Abstract

The Bacteroidales order, widely distributed among diverse human populations, constitutes a key component of the human microbiota. Members of this Gram-negative order have been shown to modulate the host immune system, play a fundamental role in the gut’s microbial food webs, or be involved in pathogenesis. Bacteria inhabiting such a complex environment as the human microbiome are expected to display social behaviors and, hence, possess factors that mediate cooperative and competitive interactions. Different types of molecules can mediate interference competition, including non-ribosomal peptides (NRPs), polyketides, and bacteriocins. The present study investigates the potential of Bacteroidales bacteria to biosynthesize class I bacteriocins, which are ribosomally synthesized and post-translationally modified peptides (RiPPs). For this purpose, 1,136 genome-sequenced strains from this order were mined using BAGEL4. A total of 1,340 areas of interest (AOIs) were detected. The most commonly identified enzymes involved in RiPP biosynthesis were radical S-adenosylmethionine (rSAM), either alone or in combination with other biosynthetic enzymes such as YcaO. A more comprehensive analysis of a subset of 9 biosynthetic gene clusters (BGCs) revealed a consistent association in Bacteroidales BGCs between peptidase-containing ATP-binding transporters (PCATs) and precursor peptides with GG-motifs. This finding suggests a possibly shared mechanism for leader peptide cleavage and transport of mature products. Notably, human metagenomic studies showed a high prevalence and abundance of the RiPP BGCs from *Phocaeicola vulgatus* and *Porphyromonas gulae*. The mature product of *P. gulae* BGC is hypothesized to display γ-thioether linkages and a C-terminal backbone amidine, a potential new combination of post-translational modifications (PTM). All these findings highlight the RiPP biosynthetic potential of Bacteroidales bacteria, as a rich source of novel peptide structures of possible relevance in the human microbiome context.

## 1. Introduction

The understanding of the human microbiome has greatly expanded in the last decades due to the accelerated evolution of -omic technologies. In 1999, experiments using molecular methods to analyze microbial communities in fecal samples (Suau et al., 1999) and subgingival crevices (Kroes et al., 1999) paved the way for the Human Microbiome Project (HMP). The HMP was a large-scale initiative launched by the National Institute of Health (NIH) in 2007 (Turnbaugh et al., 2007; Proctor et al., 2019) which, in conjunction with many other research efforts, has proven the microbiome to be an integral component of human biology. As a result, research in this field has transitioned from simply describing the human microbiome to developing a comprehensive and mechanistic understanding of the system. Moreover, the accumulated knowledge is used to advance the development of effective clinical interventions (Gilbert et al., 2018; Lynch et al., 2019). Within this new approach, bacteriocins constitute an active area of research owing to their key roles in colonization resistance, pathogenic competitive advantage, and probiotic success (Hegarty et al., 2016; García-Bayona and Comstock, 2018; Hols et al., 2019; Heilbronner et al., 2021). Bacteriocins are ribosomally synthesized and extracellularly released antimicrobial compounds of bacterial origin (Cotter et al., 2005), which can be subdivided into three classes: (i) class I or ribosomally synthesized and post-translationally modified peptides (RiPPs) with a size of less than 10kDa, (ii) class II or unmodified peptides also smaller than 10 kDa, and, (iii) class III or unmodified proteins larger than 10 kDa (Alvarez-Sieiro et al., 2016).

Previous investigations into bacteriocin production in the human microbiome have mainly examined members of the phyla Bacillota and Pseudomonadota. Less attention has been dedicated to the order Bacteroidales, the most abundant Gram-negative order in the human gastrointestinal tract (Qin et al., 2010). Besides the common gut commensal *Bacteroides* spp. (Arumugam et al., 2011), this order comprises the important periodontal pathogen *Porphyromonas gingivalis* (Mysak et al., 2014) and the ubiquitous *Prevotella* spp., which can be found in the oral cavity, gastrointestinal tract, vagina, and cow rumen (Tett et al., 2021). In spite of their prevalence, few bacteriocins are known to be produced by Bacteroidales members, most of which are class III bacteriocins. Examples of this include nigrescin, produced by *Prevotella nigrescens* (Teanpaisan et al., 2009), the wide-spread “Bacteroidales secreted antimicrobial proteins” (BSAPs; Chatzidaki-Livanis et al., 2014; Roelofs et al., 2016; McEneany et al., 2018; Shumaker et al., 2019), BcpT (Evans et al., 2022) and the eukaryotic-like ubiquitin protein (BfUbb; Chatzidaki-Livanis et al., 2017). Additionally, a group of class II bacteriocins, the broad-spectrum bacteroidetocins, has been reported to be produced by members of this order (Coyne et al., 2019). Remarkably, identification and characterization of class I bacteriocins in Bacteroidales remains elusive, raising the question whether production of RiPPs with antimicrobial activity in this order is either limited or underreported. To answer this question, this study investigates the potential production of class I bacteriocins (RiPPs) by Bacteroidales bacteria, including lanthipeptides, sactipeptides, and ranthipeptides.

Strategies for identification of novel bacteriocin producers can be broadly divided into culture- or *in silico-*based approaches. Culture-based methods have long been the standard, but *in silico* approaches have shown promise in identifying previously unknown bacteriocins (Begley et al., 2009; Donia et al., 2014; van Heel et al., 2017). Several mining tools have been developed over the years to facilitate research in this field. Popular web-based tools include BAGEL4 (van Heel et al., 2018), antiSMASH7 (Blin et al., 2023), and RODEO (Tietz et al., 2017). Additionally, the recently developed DeepRipp (Merwin et al., 2020) is another excellent and highly recommended tool to be used. Launched in 2006 and periodically updated, the BActeriocin GEnome mining tooL 4 (BAGEL4) is dedicated to the prediction of all three classes of bacteriocins based on sequence similarity to previously described ones. Furthermore, BAGEL4 takes advantage of common features and motifs present in RiPP biosynthetic gene clusters (BGCs). In most RiPP BGCs, the gene that encodes the precursor peptide, the structural gene, is positioned near accessory genes. These accessory genes are involved in post-translational modification (PTM), transport, immunity and/or regulation (extensively reviewed in Arnison et al., 2013; Bartholomae et al., 2017; Montalbán-López et al., 2021). In addition, RiPP structural genes typically exhibit two functionally distinct regions, the leader and the core peptide. While the leader peptide constitutes an N-terminal recognition region relevant for recruitment of PTM enzymes and export of the mature product, the core peptide is subjected to PTMs and, hence, is transformed into the bioactive final product (Arnison et al., 2013). Several leader peptide signatures have been described, which aid in the identification of new structural genes. Examples of these signatures include the FNLD motif of class I lanthipeptides (Plat et al., 2011) and the GG motif (Bobeica et al., 2019) present in different types of RiPPs.

The present study aims at exploring the RiPP biosynthetic capacity of Bacteroidales strains by performing an *in silico* screening using BAGEL4. From the 1,340 areas of interest (AOIs) initially detected, a subset of 9 BGCs was further investigated based on the presence of complete sets of accessory biosynthetic genes and/or frequent occurrence of the BGC across Bacteroidales genomes. The function of the genes present in the BGC was assessed, as well as the prevalence of those BGCs in metagenomic samples. Finally, a potential new combination of PTM modifications including a backbone amidine and γ-thioether linkages was hypothesized for the orally prevalent BGC of *Porphyromonas gulae* COT-052 OH1451.

## 2. Materials and methods

### 2.1. Screening of Bacteroidales genomes for putative RiPP biosynthetic gene clusters using the BAGEL4 web-server

A set of Bacteroidales genomes was obtained from Evans et al. (2022) and completed with genomes of publicly available bacterial strains in BEIresources.^1^ Genome assemblies were downloaded from NCBI using RefSeq accession numbers as of July 2022. RefSeq entries exhibiting anomalous assembly, potential contamination, or a complete absence of the genome were removed, resulting in a total of 1,136 assemblies that were used for the *in silico* screening. Supplementary Table S1 provides detailed information about the analyzed strains, including taxonomy, isolation source, and accession numbers.

To screen the selected Bacteroidales genomes for the presence of putative RiPP biosynthetic gene clusters (BGCs), the BAGEL4 web-server was used (van Heel et al., 2018).^2^ A list of the protein families or domains detected and the rules used to identify them during the screening can be found in Supplementary Table S2.

### 2.2. Functional analysis of selected RiPP BGCs

To identify promising candidate RiPP BGCs, manual curation was performed over the areas of interest (AOIs) detected by BAGEL4. The selection process involved considering the presence and arrangement of post-translational modification (PTM) enzymes, putative precursor peptides, and transporters within the AOI, as well as the occurrence of the AOI across genomes and strain isolation sources. The selected AOIs, which usually comprise over 20kbs sequence including the detected features, were manually investigated using the InterPro web-based tool (Mitchell et al., 2019)^3^ and protein–protein BLAST in the NCBI web service.

### 2.3. Metagenomic analysis of selected RiPP BGCs

The BiG-MAP bioinformatic tool (Pascal Andreu et al., 2021) was employed to explore the prevalence and abundance of selected RiPP BGCs in metagenomic samples. This tool maps shotgun sequencing reads onto BGCs predicted by antiSMASH (Blin et al., 2023). For this purpose, genomes containing selected RiPP BGCs were used as an input on antiSMASH7 in loose detection strictness, which successfully detected 6 out of the subset of 9 BGCs. The resulting antiSMASH files were used as an appropriate input for BiG-MAP. Metagenomic samples downloaded from the Human Microbiome Project (HMP^4^; RRID:SCR_004919) were used to map BGCs prevalence and abundance. A total of 808 metagenomic samples from 4 different sources (205 dorsum tongue, 201 feces, 200 gingiva, and 202 posterior fornix of vagina) were randomly selected. RiPP BGC prevalence was calculated as the ratio of HMP samples with one or more units of target BGC to the total number of HMP samples for each body source. To quantify their abundance, a RPKM (Read Per Kilobase per Million mapped reads) metric was used.

### 2.4. Sequence similarity network

The protein sequence of BGC8 YcaO (UniProt ID A0A0A2F9V0) from *P. gulae* COT-052 OH1451 was used to generate a Sequence Similarity Network (SSN) with the online Enzyme Function Initiative-Enzyme Similarity Tool (EFI-EST; Gerlt et al., 2015)^5^ using an *E* value of 5 and an alignment score (AS) of 100. The SSN for the radical S-adenosyl methionine (rSAM) enzyme (UniProt ID A0A0A2F578) was downloaded from radicalSAM.org (Oberg et al., 2022) at an AS of 45. Both SSNs were visualized using Cytoscape (Shannon et al., 2003).

### 2.5. Multiple sequence analysis

The amino acid sequence of PTM enzymes used for multiple sequence analysis (MSA) was obtained using their UniProt IDs (Supplementary Table S3) and aligned in MEGA11 (Tamura et al., 2021) using MUSCLE (Edgar, 2004) on default parameters. Jalview 2.11.2.6 (Waterhouse et al., 2009) was used for MSA visualization and Logo generation.

### 2.6. Protein sequence-structure analysis

The AlphaFold predictions (Jumper et al., 2021) for the protein 3D structures were downloaded using the enzyme’s UniProt IDs (Supplementary Table S3). When available, the X-ray structure was downloaded instead. UCSF Chimera (Pettersen et al., 2004) was used for visualization of the 3D structure and MatchMaker tool (Meng et al., 2006) for structural comparisons between proteins.

## 3. Results

### 3.1. A systematic *in silico* screening with BAGEL4 identified 1,340 RiPP areas of interest present in Bacteroidales genomes

First, the 1,136 selected Bacteroidales genomes were scanned for the presence of RiPP biosynthetic gene clusters (BGCs) using BAGEL4. This analysis yielded a total of 1,340 areas of interest (AOI) related to RiPP biosynthesis (Table 1). It should be emphasized that BAGEL4 designates an AOI upon detecting homology to a specific bacteriocin structural gene and/or certain protein motifs associated with bacteriocin BGCs (van Heel et al., 2018). Therefore, AOI hits should be considered an indication of minimal potential RiPP production, rather than the actual presence of a BGC. Detailed information about the list of post-translational modification (PTM) enzyme families or domains detected and the rules used to identify them can be found in Supplementary Table S2.

**Table 1:** Areas of interest (AOIs) detected by BAGEL4 for different Bacteroidales families.

			Areas of interest (AOIs)																
Family	Amount of genomes	Total AOI	rSAM	rSAM + LanC	rSAM + GlyS	rSAM + LanC + GlyS	rSAM + YcaO	rSAM + YcaO + GlyS	rSAM + ThioE	rSAM + YcaO + ThioE	ThioE	ThioE+ YcaO	LanC	LanD	LanM	LanM + LanC	GlyS	LasC	ComX1
*Bacteroidaceae*	623	911	578	205	1	16	7	1	0	2	76	2	17	1	0	0	0	0	5
*Balneicellaceae*	1	0	0	0	0	0	0	0	0	0	0	0	0	0	0	0	0	0	0
*Barnesiellaceae*	3	2	2	0	0	0	0	0	0	0	0	0	0	0	0	0	0	0	0
*Dysgonomonadaceae*	13	5	5	0	0	0	0	0	0	0	0	0	0	0	0	0	0	0	0
*Lentimicrobiaceae*	1	1	1	0	0	0	0	0	0	0	0	0	0	0	0	0	0	0	0
*Muribaculaceae*	9	9	5	0	0	0	0	0	1	0	0	0	3	0	0	0	0	0	0
*Odoribacteraceae*	28	18	13	1	0	0	1	0	0	0	3	0	0	0	0	0	0	0	0
*Paludibacteraceae*	2	1	0	0	0	0	0	0	0	0	0	0	0	0	0	0	0	1	0
*Porphyromonadaceae*	119	52	40	1	0	0	5	2	0	0	0	0	3	1	0	0	0	0	0
*Prevotellaceae*	195	174	152	2	0	0	1	0	0	4	2	8	3	0	1	1	0	0	0
*Rikenellaceae*	35	10	10	0	0	0	0	0	0	0	0	0	0	0	0	0	0	0	0
*Tannerellaceae*	106	155	52	54	6	25	1	0	2	0	4	0	9	0	0	0	2	0	0
*Tenuifilaceae*	1	2	0	0	0	0	0	0	0	0	1	0	1	0	0	0	0	0	0
*Williamwhitmaniaceae*	1	0	0	0	0	0	0	0	0	0	0	0	0	0	0	0	0	0	0
TOTAL	1,136	1,340	858	263	7	41	15	3	3	6	86	10	36	2	1	1	2	1	5

See also Supplementary Table S4 for the full data set of designated AOIs per strain. rSAM, radical SAM; LanC, Lanthionine synthetase C-like; GlyS, Peptide S-glycosyltransferase; YcaO, YcaO domain containing protein; ThioE, Dehydrogenase; LanD, Decarboxilase; LanM, Type II lanthipeptide biosynthesis protein; LasC, Macrolactam synthetase.

**Figure 1:**
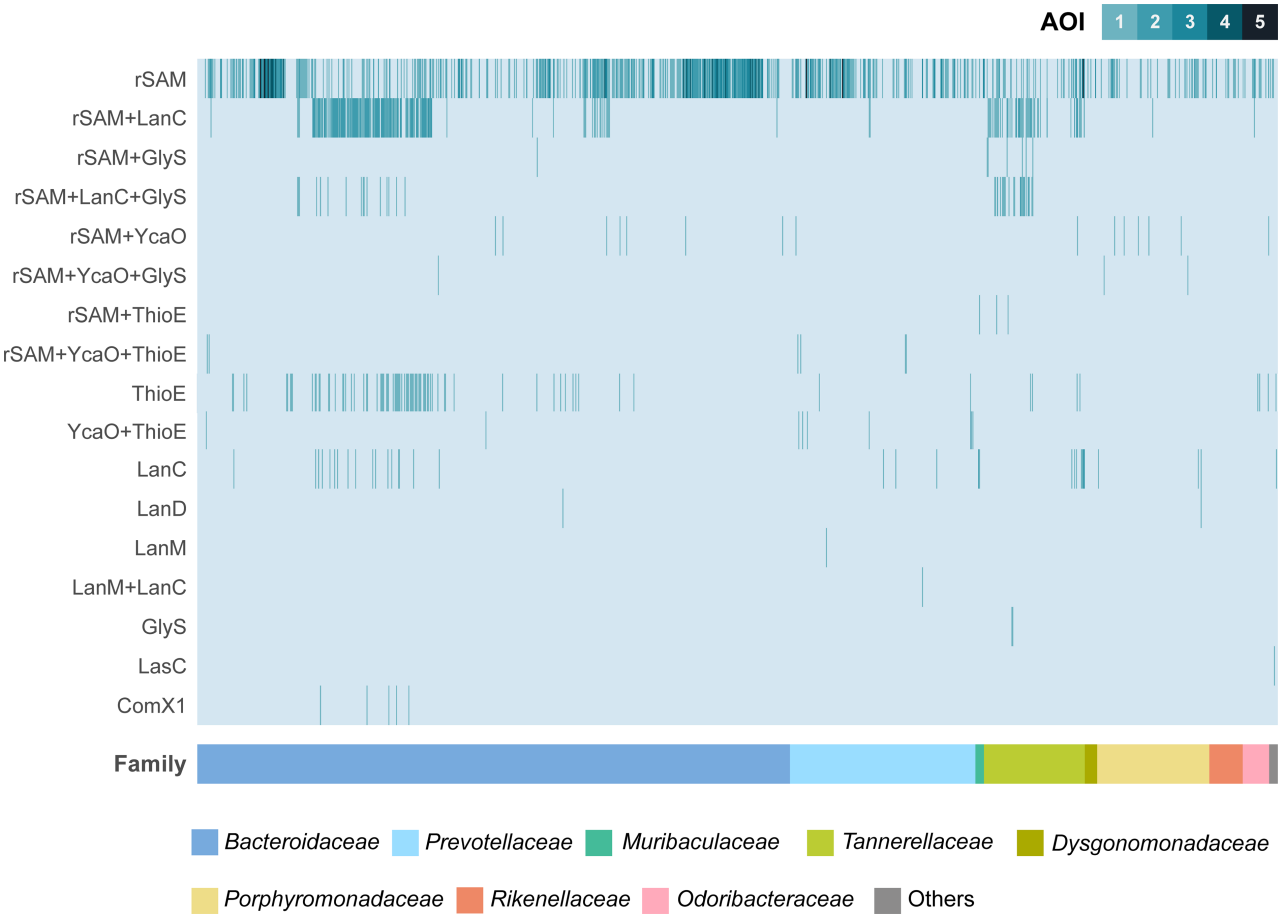
Overview of areas of interest (AOIs) detected by BAGEL4 in the set of 1,136 Bacteroidales genomes. The bacterial strains are ordered according to their Family. Families with less than 5 genomes were clustered under “Others” category for visualization purposes (*Balneicellaceae, Barnesiellaceae, Lentimicrobiaceae, Paludibacteraceae, Tenuifilaceae, Williamwhitmaniaceae*). rSAM, radical S-adenosylmethionine; LanC, Lanthionine synthetase C-like; GlyS, Peptide S-glycosyltransferase; YcaO, YcaO domain containing protein; ThioE, Dehydrogenase; LanD, Decarboxilase; LanM, Type II lanthipeptide biosynthesis protein; LasC, Macrolactam synthetase.

The most frequently identified AOIs in this analysis contain one or more radical S-adenosylmethionine (rSAM) genes, either alone or in combination with other genes commonly involved in RiPP BGCs (Figure 1; Table 1). AOIs containing rSAM as the sole biosynthetic enzyme are widely distributed among the different Bacteroidales families. In fact, the only families that did not exhibit rSAM containing AOIs were those underrepresented in the analysis, such as *Tenuifilaceae* or *Paludibacteraceae*. The rSAM superfamily represents one of the largest and most functionally-diverse enzyme superfamilies (Oberg et al., 2022). Through radical-mediated reactions, rSAM enzymes are able to perform complex chemical transformations and rearrangements, dehydrogenations, methylations, sulfur insertions, and modifications of DNA, RNA and peptides (Sofia et al., 2001; Shisler and Broderick, 2012). Enzymes of this superfamily have been shown to participate in the biosynthesis of various classes of RiPPs, carrying a wide array of reactions, including epimerization, thioether crosslinking, and methyltransference activity (Montalbán-López et al., 2021).

Following single rSAM AOIs, genetic regions containing both rSAM and LanC genes are particularly abundant. LanC enzymes are part of class I lanthipeptides BGCs and they install (methyl)lanthionine rings between the C_β_ atom of dehydrated serine or threonine residues and the sulfhydryl group of cysteine residues (Lubelski et al., 2008; Repka et al., 2017). These hybrid rSAM + LanC AOIs are notably frequent in strains of *Bacteroides fragilis, Bacteroides thetaiotaomicron, Parabacteroides distasonis,* and *Tannerella forsythia* (Supplementary Table S4). In some *B. fragilis* and *P. distasonis* strains, a combination of rSAM, LanC, and a gene containing a peptide S-glycosyltransferase (GlyS) domain is detected. rSAM genes can also be found in other combinations, including rSAM + YcaO. YcaO proteins have been shown to participate in several RiPP BGCs via azoline, thioamide, or amidine formation (Burkhart et al., 2017; Clark and Seyedsayamdost, 2022). Areas exhibiting rSAM + YcaO are particularly abundant in strains of the genus *Bacteroides* and *Porphyromonas.*

Stand-alone LanC cyclase genes were detected in several strains from different Bacteroidales families, with special prevalence in *B. fragilis* and *T. forsythia* strains. Consecutive action of LanB and LanC is required for lanthionine ring installation in class I lanthipeptides (Arnison et al., 2013) and, intriguingly, none of the screened genomes contained LanB dehydratase genes. In contrast, two AOIs exhibiting LanM-like genes were detected in *Prevotella* strains, either stand-alone in *P. buccalis* DNF00985 or in combination with a LanC-like gene in *P. multisaccharivorax* DSM 17128 (Figure 1; Supplementary Table S4). LanM enzymes participate in the biosynthesis of class II lanthipeptides, carrying out both dehydration and cyclization steps (Arnison et al., 2013).

Remarkably, experimentally described RiPPs were mostly absent from the *in silico* screening, with the exception of ComX. Five strains of *B. fragilis* were found to possess a protein exhibiting homology to the structural gene of the *Bacillus amyloliquefaciens* JRS8 quorum sensing pheromone ComX. Further exploration revealed that while the translated gene showed partial homology to the pheromone precursor, the key tryptophan residue was absent in the *B. fragilis* gene (data not shown). This tryptophan is a fully conserved residue whose post-translational modification results in the formation of a tricyclic structure (Okada et al., 2005). In light of this consideration and due to the unusual large size of the *B. fragilis* gene, ComX matches were deemed as false positives and, hence, not pursued any further.

### 3.2. Further analysis of putative RiPP biosynthetic gene clusters

**Figure 2:**
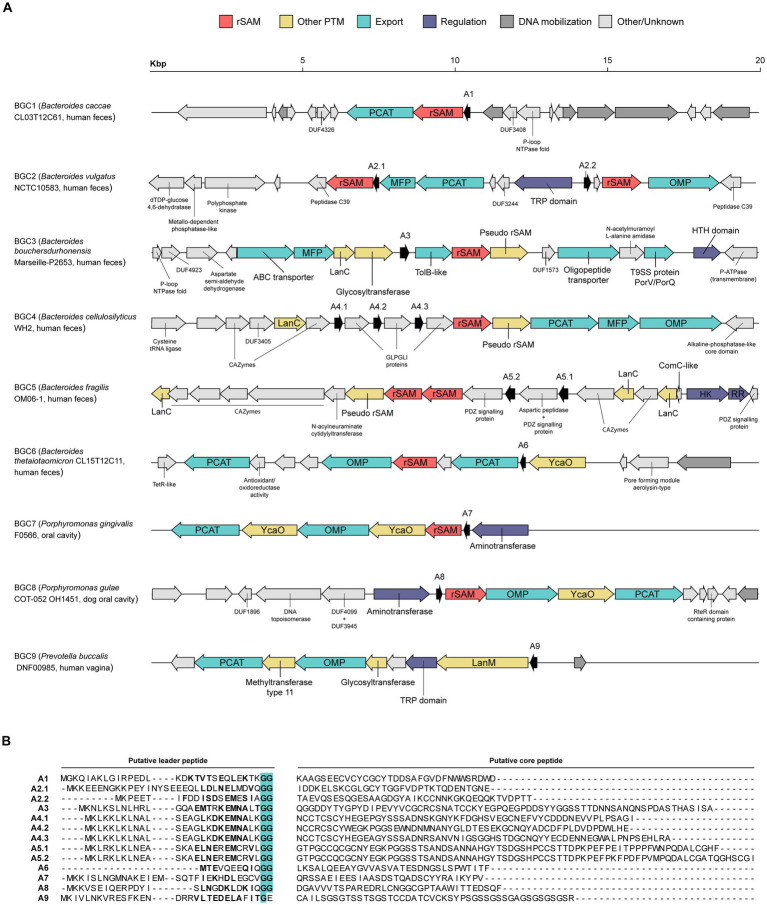
Selected subset of RiPP biosynthetic gene clusters (BGCs) detected on Bacteroidales strains. **(A)** Diagrammatic representation of RiPP BGC candidates. The label of each BGC indicates the putative producer and its isolation source of origin. Putative precursor peptides are designated with an “A” and the number of their corresponding BGC. **(B)** Translated sequence of the putative precursor peptides and the predicted division between leader and core peptide. Leader peptide residues appearing on a specific position of the GG-motif (TIGR01847) with a probability ≥ 0.09 are highlighted in bold. The canonical doubly Gly that gives name to this motif is highlighted in cyan. PCAT, peptidase-containing ATP-binding transporter; rSAM, radical S-adenosylmethionine; MFP, membrane fusion protein; OMP, outer membrane protein; TRP, tetratricopeptide-like helical repeat; HK, histidine protein kinase; RR, response regulator protein.

Since the BAGEL4 analysis gave a high frequency of AOIs containing orphan enzymes (data not shown), a manual curation process of the AOIs was employed. This second round of selection focused on the presence and arrangement of PTM enzymes, putative precursor peptides, and transporters within the AOI. Additionally, the occurrence of the gene cluster across different genomes and the strain isolation source were given consideration. As a result, 9 putative interesting RiPP BGCs were selected and functionally annotated (Figure 2A). Due to their already reported abundance, it is unsurprising that 8 out 9 of the BGCs contain at least a rSAM enzyme. The PTM composition varies among the set of BGCs, as some clusters feature only rSAM enzymes (BGC1,2), whereas others exhibit a combination of rSAM and other types of PTM enzymes such as LanC (BGC3,4,5) or YcaO (BGC6,7,8). Only the last selected BGC is characterized by the absence of a rSAM and the presence of a LanM enzyme (BGC9), an enzyme involved in class II lanthipeptide biosynthesis and mostly found in Gram-positive bacteria. Strikingly, BGC3,4,5 contain LanC cyclases but lack the LanB encoded proteins, necessary for class I lanthipeptide biosynthesis. LanC cyclases are known to show highly conserved sequence features, namely a triad of residues involved in Zinc ion coordination (C-gap-CH) and additional single residues involved in active site formation (Li et al., 2006; Marsh et al., 2010). However, the putative LanC sequences identified in BGC3,4,5 do not show these motifs (Supplementary Figure S1).

Additional accessory enzymes were detected in several BGCs, which have the potential to introduce PTMs. These enzymes include pseudo rSAMs (BGC3,4,5), glycosyltransferases (BGC3,9), aminotransferases (BGC7,8) and a methyltransferase (BGC9). Although no specific function has been experimentally assigned to pseudo rSAMs, InterPro reports that members of this family (IPR026418) co-occur with rSAM enzymes and a precursor peptide, as it is observed in BGC3,4,5 (Figure 2A). It has been hypothesized that these pseudo rSAM are likely working in partnership with the rSAM to introduce chemical modifications in the putative precursor peptide.

Regarding export systems, all BGCs with the exception of BGC3 and BGC5 encompass at least one gene encoding a C39 peptidase-containing ATP-binding transporter (PCAT). Transport by PCAT containing systems is a common strategy for leader peptide cleavage and export of the mature compound out of the cell (Havarstein et al., 1995; Montalbán-López et al., 2021). Most of the precursor peptides annotated exhibit residues frequently present on a GG motif (TIGR01847; Figure 2B), a signature for peptides subjected to PCAT leader cleavage and export (Arnison et al., 2013; Bobeica et al., 2019). Notably, precursor peptides associated to BGC3 and BGC5 contain several residues matching the GG motif signature despite lacking a PCAT in their BGCs.

Other Gram-negative bacteria have been shown to employ PCAT in the translocation of antimicrobial peptides. A well-known example is the class II bacteriocin colicin V (ColV) export system, which requires PCAT, a membrane fusion protein (MFP), as well as the outer membrane protein (OMP) TolC for crossing both inner and outer membranes (Gilson et al., 1990; Wu and Tai, 2004). The ColV structural gene is located within the same gene cluster as the PCAT and MFP genes, whereas TolC is encoded elsewhere (Gilson et al., 1990). In the analyzed BGCs, several genetic arrangements are found (Figure 2A). For instance, BGC4 encodes all necessary genes for peptide export in Gram-negatives, featuring PCAT, MFP, and OMP genes. BGC2 also possesses a complete set of transporter genes, although unlike BGC4, the OMP gene is not encoded in tandem with PCAT and MFP. Notably, BGC6,7,8 and 9 contain both PCAT and OMP genes, yet no MFP was identified. This raises the question whether the transport for their mature compounds is governed by a distinct mechanism or if the detection of an MFP remains to be accomplished. The remaining BGCs possess different lay-outs, including only a PCAT in BGC1, several types of transporters in BGC3, or the total absence of them in BGC5.

Besides PTM and transporter genes, RiPP BGCs usually contain other complementary genes that are involved in self-immunity and regulation of expression. Before or during manual curation, no proteins displaying homology with already described immunity proteins were detected. Nonetheless, it has been demonstrated that Bacteroidetocins, unmodified antimicrobial peptides produced by members of the Bacteroidales order, can inhibit the growth of the producer strain (Coyne et al., 2019). This observation suggests that it may be possible to produce RiPPs with antimicrobial activity in the absence of immunity proteins. On the other hand, some efflux systems that are present in selected BGCs could be involved in self-immunity as reported for the antimicrobial peptides microcin J25 (MccJ25; Bountra et al., 2017) and microcin B17 (MccB17; Garrido et al., 1988), which are produced by other Gram-negative bacteria. Immunity to MccJ25 is achieved using the same ABC exporter, opening the possibility of a dual role for PCAT exporters in selected BGCs. MccB17 and ColV exhibit dedicated transporters for immunity and, therefore, the different transporters in BGC3 could be involved in immunity.

In contrast to a notable small detection rate of self-immunity genes, several potentially regulatory proteins were detected. This includes a helix-turn-helix (HTH) domain containing gene in BGC3 and a signal transduction system in BGC5, composed of a histidine kinase (HK) and a response regulator (RR). Both types of systems have been shown to be involved in transcriptional regulation (Gallegos et al., 1993; Kuipers et al., 1998; West and Stock, 2001). Additionally, tetratricopeptide-like helical (TRP) domain-encoding genes appear in BGC2 and 9. TRP domains are involved in protein–protein interaction and, among their many biological functions, have been suggested to play a role in gene expression regulation in other bacteria after pheromone binding (Capodagli et al., 2020).

BGC7 and 8 possess a gene containing an N-terminal aminotransferase domain (IPR004839) followed by a homeodomain (IPR009057). Aminotransferase involvement in RiPP biosynthesis has been previously described for methanobactocins (Mbns), copper-chelating peptides firstly identified in methanotrophic bacteria (Kim et al., 2004; Park et al., 2018). However, the homeodomain found in BGC7 and 8, a known DNA-binding motif, points toward their role as transcription factors. Interestingly, a similar combination of domains has been described for the *Bacillus subtilis* transcription factor GabR (Belitsky and Sonenshein, 2002), where the homeodomain is situated N-terminally and the aminotransferase C-terminally.

Finally, BGC5 shows an interesting cluster architecture. It presents two putative precursor peptides with a predicted long core peptide sequence, each of them followed by a putative signaling protein (Figures 2A,B). Although it appears to lack transporter sequences, BGC5 presents two rSAM, one pseudo rSAM and three LanC-like sequences, which might not perform the classical LanC activity, as previously discussed (Supplementary Figure S1). Finally, two sets of CAZymes complete the distinguishing features of BGC5. While it could be hypothesized that this cluster, and related clusters from *B. fragilis* species, could be a source of novel antimicrobials, no antimicrobial activity can be concluded from this analysis.

### 3.3. Prevalence and abundance of selected RiPP BGCs on human metagenomes

**Figure 3:**
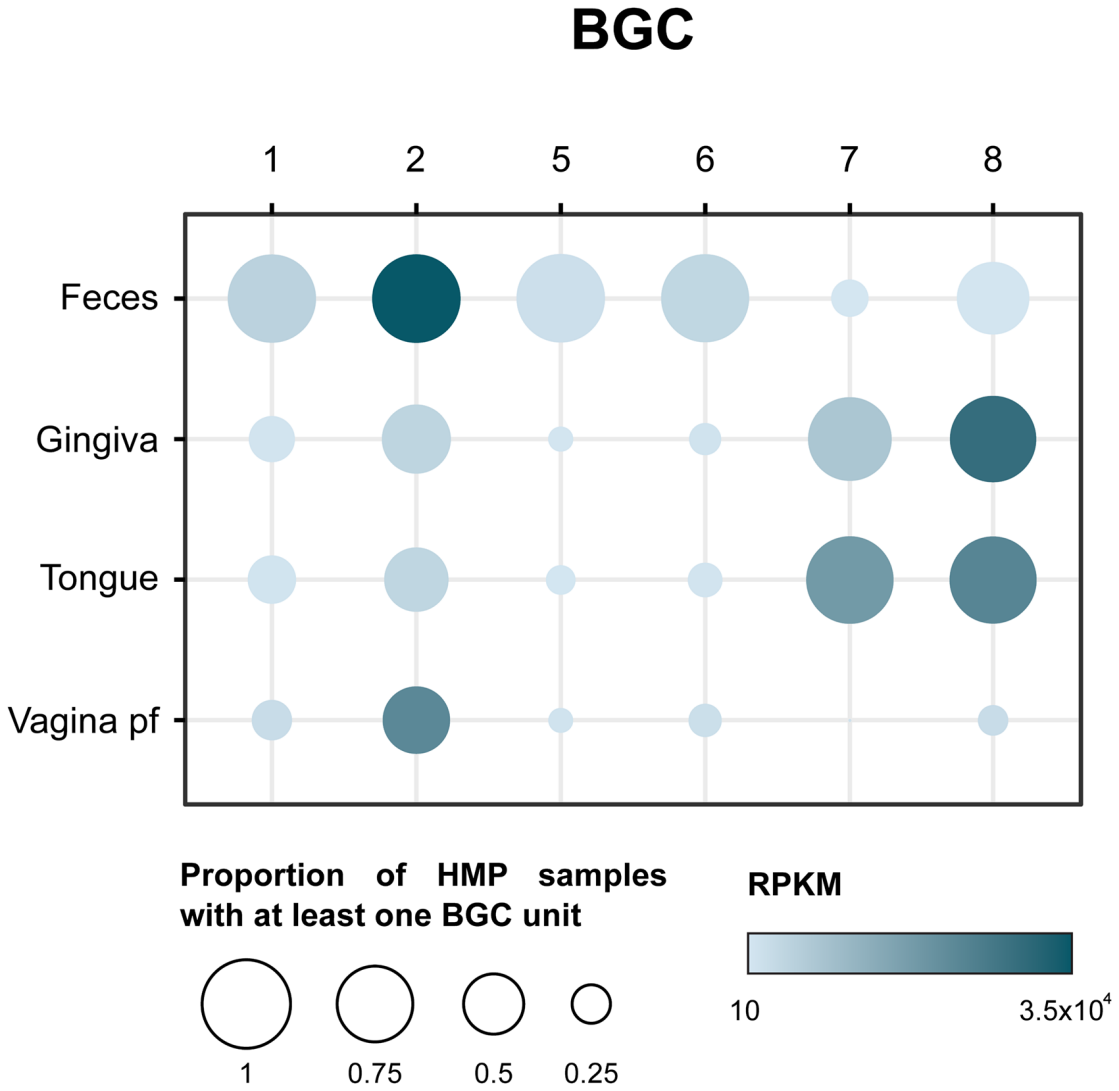
Balloon plot showing the prevalence and abundance of selected BGCs in Human microbiome project (HMP) samples from four different sources: feces, gingiva, dorsum tongue, and posterior fornix of the vagina. Prevalence is shown as the proportion of HMP samples with at least one BGC unit to the total number of samples per source. Abundance is expressed in reads per kilobase per million mapped reads (RPKM).

The microbiome has been found to be crucial for human health, and the production of antimicrobial peptides can have a significant impact on the diversity and function of the microbiome. To assess the potential significance of the RiPP BGCs detected in the human microbiome, the prevalence and abundance of those BGCs were studied on metagenomic samples from the Human Microbiome Project (HMP). For that purpose, a total of 808 metagenomic samples from 4 different body sources (feces, dorsum tongue, gingiva, and posterior fornix of vagina) were analyzed using BiG-MAP (Pascal Andreu et al., 2021). Out of the 9 selected BGCs, 6 were used as input. The presence of those BGCs in feces concurs with the isolation place of their producer strains (Figure 2A). As seen in Figure 3, BGC1, 2, 5 and 6 are present in almost all fecal samples. BGC7 and 8, only detected in *Porphyromonas* strains that were isolated from dog oral cavities, are surprisingly prevalent in human gingiva and tongue samples. As could be expected from their isolation source, the prevalence of the selected RiPP BGCs in vaginal samples is lower compared to other sources. Only BGC2, prevalent in every body source, appears in more than half of the vaginal samples analyzed.

Regarding their abundance, expressed in read per kilobase per million (RPKM), BGC8 was the most abundant cluster in both gingiva and on the tongue, while BGC2 showed the highest abundance in fecal samples. Despite being present just in around half of the vaginal samples, BGC2 is quite abundant in that body source, reaching 2.2 × 10^4^ RPKM. The opposite takes place in fecal samples with BGC1,5 and 6, whose abundance is low in spite of being quite prevalent on this body source.

### 3.4. Characterization of the orally prevalent BGC from *Porphyromonas gulae* COT-052 OH1451

**Figure 4:**
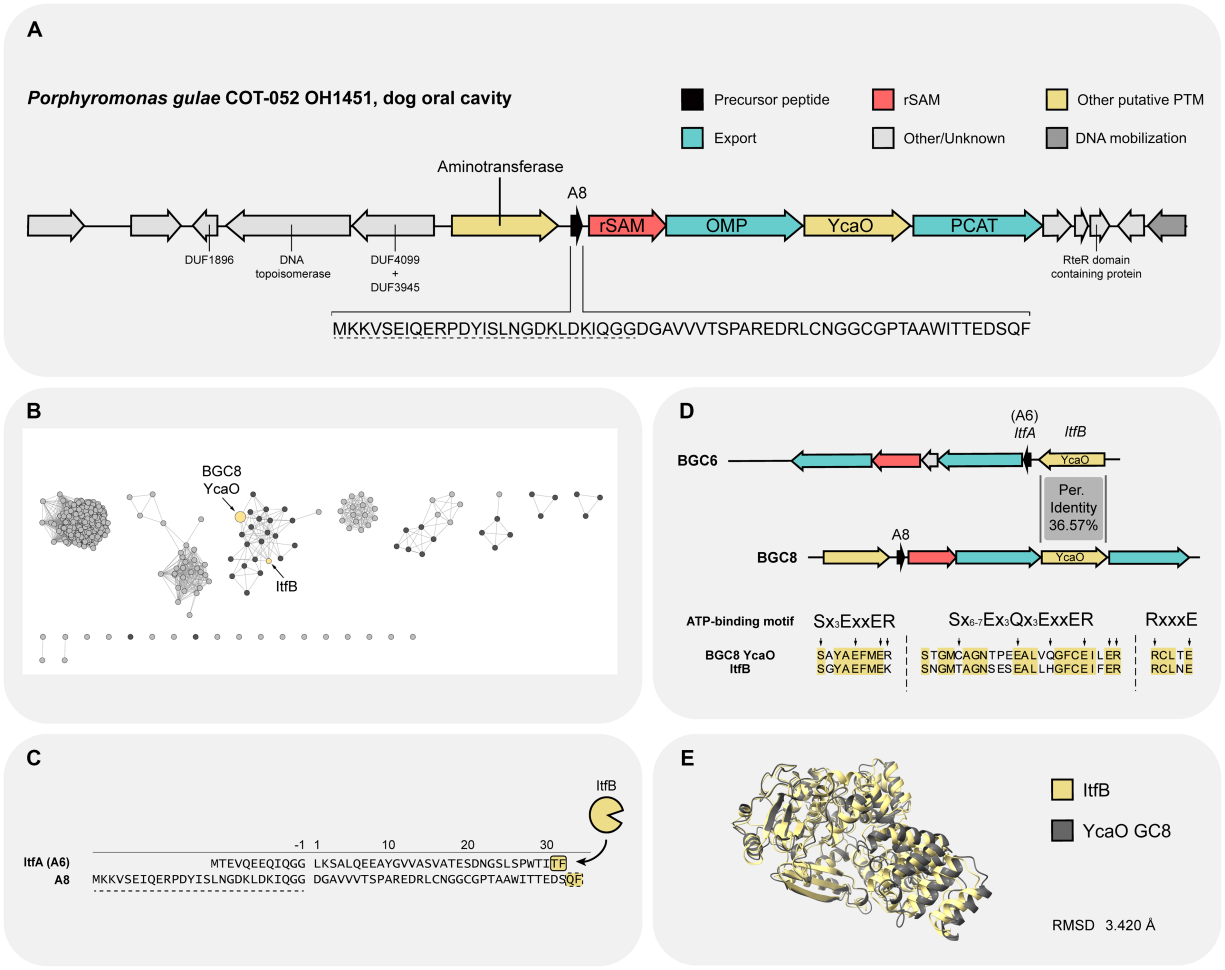
Overview of *Porphyromonas gulae* COT-052 OH1451 BGC and its YcaO PTM enzyme. **(A)** Diagrammatic representation of the BGC with annotated biosynthetic and neighboring proteins. The sequence of the precursor peptide (A8) is shown below the scheme and the residues constituting the leader peptide are underlined with a dashed line. **(B)** Sequence similarity network (SSN) generated using the amino acid sequence of YcaO BGC8. An E value of 5 and an alignment score (AS) of 100 were used to generate the SSN. Retrieved proteins encoded by Bacteroidetes bacteria are highlighted in black. **(C)** Precursor peptide sequence of both ItfA (A6 from BGC6) and A8 from BGC8. Putative leader peptides are highlighted using a dashed line. Target residues for ItfB PTM modification are highlighted in yellow (Clark and Seyedsayamdost, 2022). Putative residues involved in PTM modification by YcaO BGC8 are highlighted in yellow with a dashed border. **(D)** Protein alignment between YcaO from BGC8 and the one of BGC6 (ItfB) regarding the ATP binding motif described in Dunbar et al. (2014). Conserved residues between the two proteins are highlighted in yellow. Residues signature of the ATP-binding are highlighted with arrows. **(E)** Structure superimposition of ItfB and YcaO BGC8. It resulted in a root mean square distance (RMSD) of 3.420 for all pairs of atoms. PCAT, peptidase-containing ATP-binding transporter; rSAM, radical S-adenosylmethionine; OMP, outer membrane protein; RMSD, root-mean-square deviation.

Further characterization of the different components of BGC8 was undertaken due to its prevalence and abundance in oral metagenomic samples from human origin (Figure 3). The cluster is characterized by the presence of a YcaO and a rSAM (Figure 4A). Additionally, a small ORF of 61 residues is suspected to be the precursor peptide. The presence of an N-terminal bacteriocin type GG-signal sequence (TIGR01847) matches the presence of a PCAT export system (Arnison et al., 2013; Bobeica et al., 2019). Therefore, it is predicted that the leader peptide accounts for the first 27 residues.

In order to gain more insight into the YcaO enzyme function, its amino acid sequence was used to generate a sequence similarity network (SSN) with the online Enzyme Function Initiative-Enzyme Similarity tool (EFI-EST; Figure 4B). According to the SSN analysis, the YcaO enzyme from BGC8 clusters together with the YcaO from BGC6, which was experimentally characterized and named ItfB by Clark and Seyedsayamdost (2022) during the course of this study. ItfB was shown to be able to convert the backbone amide into an amidine between the last two C-terminal residues Thr and Phe, a new type of modification for the YcaO family (Clark and Seyedsayamdost, 2022). In spite of having low sequence similarity, the precursor peptides from both BGCs, A8 and A6 (ItfA), have as a final residue a Phenylalanine (Figure 4C). Further exploration of the similarity between the two YcaO enzymes showed that both enzymes share key residues for the ATP-binding motif typical of YcaO domains (IPR003776; Dunbar et al., 2014; Figure 4D). Although the sequences of these enzymes have a low percentage of identity, 36.57%, sequence-structure analysis revealed a similar protein structure for both enzymes (Figure 4E). These findings suggest that the YcaO enzyme from BGC8 may have a similar mode of action to ItfB, installing a backbone amidine on A8 between Gln33 and Phe34.

**Figure 5:**
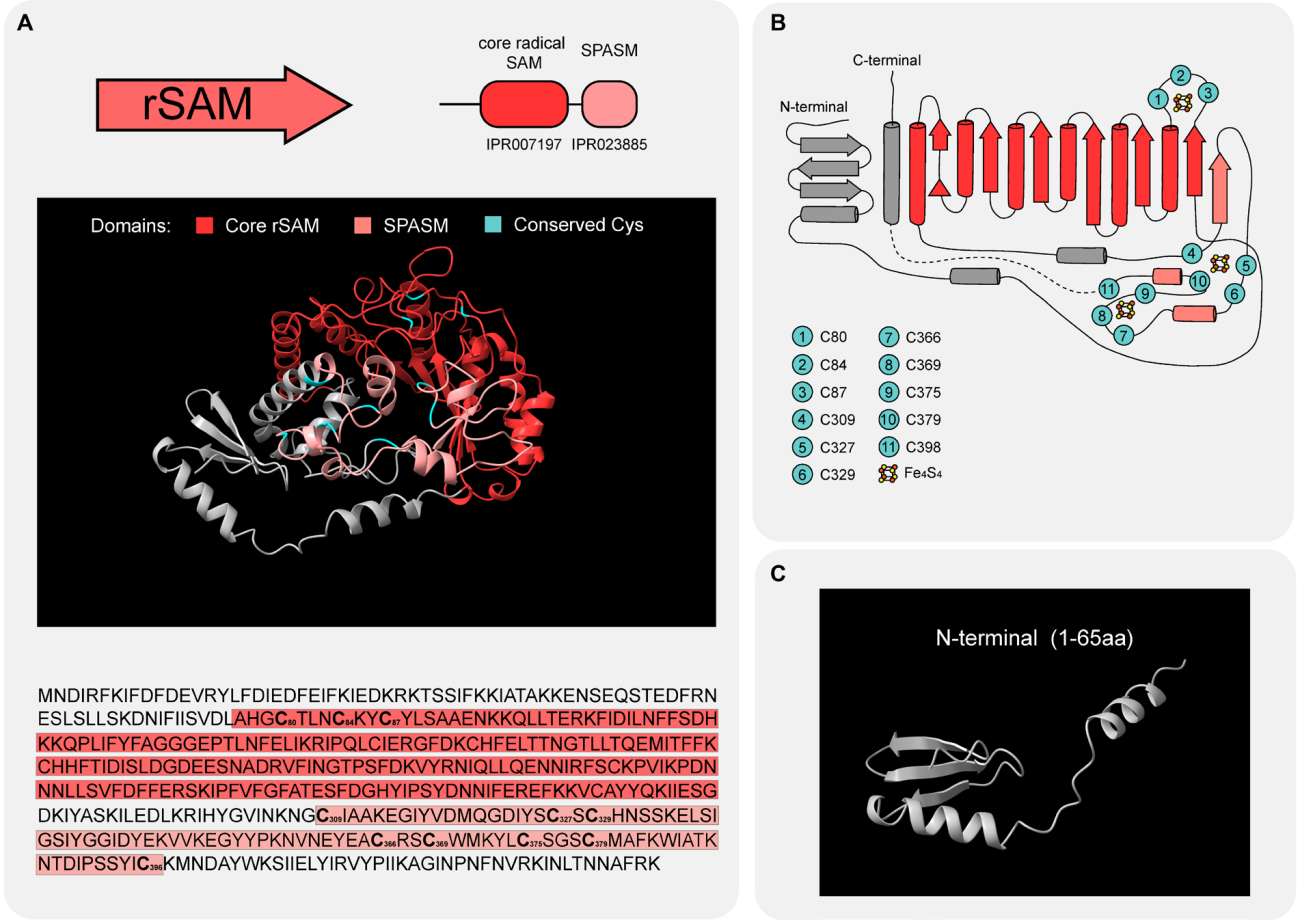
rSAM BGC8 structure. **(A)** The overall predicted structure (up) and amino acid sequence (down) of rSAM BGC8. The β_6_/α_6_ core domain (IPR007197; red) contains one [4Fe-4S] cluster that coordinates a molecule of SAM. The C-terminal SPASM domain (IPR023885; pale red) is hypothesized to contain the [4Fe-4S] clusters AuxI and AuxII. Relevant Cys residues are highlighted in cyan in the predicted structure and in bold in the amino acid sequence. **(B)** Interpretation of rSAM BGC8 topology using the AlphaFold prediction (UniProt ID A0A0A2F578) and the superimposition of rSAM BGC8 3D structure with the one of CteB (PDB 5WGG). β strands are illustrated as arrows and α helices as cylinders. Cys suspected to play a role in [4Fe-4S] cluster coordination are highlighted in cyan. For representation purposes, the dashed line connects Cys398 and the last α helix which are not separated in space on the 3D structure. **(C)** N-terminal domain with a structure reminiscent of RiPP recognition elements (RRE).

The rSAM enzyme of BGC8 contains a core rSAM domain (IPR007197), the unifying structural theme of the diverse rSAM superfamily (Grell et al., 2015). This domain adopts a partial triose-phosphate isomerase (TIM) barrel conformation (Nicolet and Drennan, 2004) and possesses a conserved CX_3_CX_2_C motif (Sofia et al., 2001). The TIM-barrel fold consists of a sixfold repeat of (βα) units, such that six parallel β-strands on the inside are covered by six α-helices on the outside. The CX_3_CX_2_C motif is located in a loop between strand β_1_ and helix α_1_ (Nicolet and Drennan, 2004). Mechanistically, the three cysteinyl sulfur groups of this motif coordinate three iron ions from a [4Fe-4S] cluster and the remaining iron anchors the SAM (Walsby et al., 2002). Additionally, an aromatic residue before the third cysteine of the CX_3_CX_2_C motif is suggested to be involved in SAM coordination via hydrophobic interactions (Grell et al., 2015). In Figures 5A,B, AlphaFold predicts that the rSAM enzyme of BGC8 adopts a partial TIM barrel conformation despite the lack of a complete β_6_ strand. The CX_3_CX_2_C motif, present as “C_80_TLNC_84_KYC_87_,” is located on the loop between strand β_1_ and helix α_1_. The Tyr in from of Cys87 is the aromatic residue suspected to be involved in SAM coordination.

Beyond their core domain, rSAM enzymes display a great diversity in structure, with functionalized N- and C-terminal extensions. N-terminal extensions involved in leader peptide recognition have been previously described for rSAM enzymes involved in peptide modification (Wieckowski et al., 2015; Grove et al., 2017). Named RiPP precursor recognition element (RRE), this N-terminal extension displays three α-helices and three β-sheets in a wHTH motif (Montalbán-López et al., 2021). The rSAM of BGC8 presents a slightly shorter extension with one α-helix and three β-sheets (Figure 5C), reminiscent of the canonical RRE described for other PTM enzymes. As a C-terminal domain, this enzyme exhibits a SPASM domain (IPR023885) that can bind two auxiliary [4Fe-4S] clusters via seven or eight cysteine residues (Haft and Basu, 2011). Enzymes containing this additional domain usually participate in post-translational modification of peptides (Grell et al., 2015). The role of the two auxiliary [4Fe-4S] clusters in RiPP modification has been suggested to be system dependent (Mendauletova et al., 2022), with an inter-Cys residue spacing varying among enzymes (Oberg et al., 2022). In Figures 5A,B, cysteine residues suspected to be involved in the binding of three [4Fe-4S] clusters- one involved in SAM coordination and two auxiliary ones- are highlighted in cyan, suggesting the role of BGC8 rSAM in peptide modification.

To elucidate whether the rSAM present in BGC8 and BGC6 (ItfD) could be catalyzing similar reactions, the new web-based resource RadicalSAM.org was used (Oberg et al., 2022). Using SSNs, RadicalSAM.org attempts to enable identification of isofunctional groups of rSAM enzymes. Exploration of the SSN generated for the rSAM superfamily showed that rSAM BGC8 and ItfD are both clustered together in Megacluster 1-1. SPASM domain containing enzymes cluster together in this Megacluster (Oberg et al., 2022), which matches the detection of such domain in rSAM BGC8 and ItfD. Nevertheless, a higher alignment score (AS) clusters the rSAM from BGC8 together with QhpD and the ranthipeptide maturases CteB and Tte1186 (Supplementary Figure S2A; Nakai et al., 2015; Bruender et al., 2016; Grove et al., 2017). Ranthipeptides subjected to CteB and Tte1186 maturases are RiPPs characterized by the presence of γ-thioether linkages between Cys and Thr residues, specifically Thr for CteA and Tte1186a. The presence of two Cys and Thr residues on the precursor peptide (Figure 4A) makes plausible that the rSAM from BGC8 performs such thioether linkages. In addition, multiple sequence alignment (Supplementary Figure S2B) and sequence-structure alignment (data not shown) shows a similar inter-Cys residue spacing between CteB and the rSAM BGC8. Notably, QhpD, CteB, and Tte1186 display 7 Cys residues on their SPASM domain, while the rSAM from BGC8 possesses a total of 8 (Supplementary Figure S2B).

## 4. Discussion

Bacteroidales bacteria are common members of human-associated microbiota (Arumugam et al., 2011; Mysak et al., 2014; Tett et al., 2021), yet their capacity for class I bacteriocin (RiPPs) production is largely unknown. This study sheds light on the RiPP biosynthetic potential of members of this order by identifying potential RiPP BGCs, examining the prevalence of a subset of BGCs in metagenomic samples, and discussing some of their biosynthetic enzymes. The databases of detected RiPP BGCs (see Supplementary Tables S4, S5) constitute valuable resources for future research aimed at discovering new RiPPs and their significance in the human microbiome. While useful, the approach utilized in this study is not without limitations. The ability to detect RiPP BGCs is limited by the identification criteria established in the current version of BAGEL4. Considering the continuous discovery of new RiPP classes and features, a future update of BAGEL or using an alternative prediction tool could complement the findings described in this study.

The *in silico* screening with BAGEL4 described in this paper shows that the most frequent AOIs contain only a rSAM enzyme (Figure 1; Table 1). The rSAM superfamily is one of the largest and most functionally diverse enzyme superfamilies (Oberg et al., 2022). Enzymes of this superfamily are able to perform complex chemical transformations in a wide array of substrates, including DNA, RNA, and peptides (Sofia et al., 2001; Shisler and Broderick, 2012). Therefore, given the multifaceted nature of this superfamily, establishing a definitive association between the sole detection of a rSAM gene and actual peptide PTM remains a challenge. To solve this issue, further investigation using additional resources was conducted. The utilization of recently developed tools, such as radicalSAM.org (Oberg et al., 2022) or AlphaFold predictions (Jumper et al., 2021), has proven its usefulness in generating hypothesis for the functional assignment of the *Porphyromonas gulae* rSAM in BGC8.

LanC enzymes conform the second most commonly detected PTM enzyme in this study, either stand-alone or in combination with other PTM enzymes. Despite their frequency, they were never observed together with LanB enzymes (Figure 1; Table 1). A closer examination of the manually curated clusters (Figure 2), reveals that several of these LanCs appear in the vicinity of Carbohydrate-Active Enzymes (CAZymes). Coupled with the lack of key residues for LanC cyclase function (Supplementary Figure S1), it is likely that these proteins are more similar to endogluconases and not involved in lanthipeptide BGCs (Li et al., 2006; Walker et al., 2020). In contrast, LanB enzymes (dehydratases) were completely absent in Bacteroidales genomes, which is in accordance with previous findings (Walsh et al., 2015). Despite recent reports suggesting the Bacteroidetes phylum as a rich source of class I lanthipeptides (Caetano et al., 2020; Walker et al., 2020), the majority of LanB-encoding genomes are limited to orders other than Bacteroidales. Moreover, in this study just two genomes possessed the LanM enzyme required for class II lanthipeptide biosynthesis. Based on the available information, lanthipeptide biosynthesis is not a common trait found in Bacteroidales bacteria.

Further analysis of a subset of RiPP BGCs revealed precursor peptides possessing residues frequently present in a GG motif (Figure 2B), which are usually accompanied by a corresponding PCAT. The involvement of PCATs in leader peptide cleavage and export of the mature product is a common mechanism for precursor peptides containing a GG-motif (Arnison et al., 2013; Montalbán-López et al., 2021). In addition, a similar association has been previously reported in bacteroidetocins, class II bacteriocins produced by Bacteroidales bacteria (Coyne et al., 2019). Bacteroidetocins are predicted to have a leader peptide cleaved after a GG motif and their BGCs contain a PCAT. Taken together, this suggests the use of PCAT for leader peptide cleavage and export as a common mechanism in bacteriocin biosynthesis in Bacteroidales bacteria.

The study of the representation of BGCs in metagenomic samples, when possible, provided a first indication of their relevance in the human microbiome. BGC2 of a *Phocaeicola vulgatus* strain [recently reclassified from *Bacteroides vulgatus* (García-López et al., 2019)], appears to be prevalent in different body sources and especially abundant in fecal samples (Figure 3). It is notable that BGC8 was well represented in human samples too, in spite of only being originally detected on a *P. gulae* strain isolated from a dog’s oral cavity. However, to provide conclusive evidence that these BGCs are produced in humans, additional experiments are necessary. As a first step, indirect detection of the BGCs on metatranscriptomic reads would provide insight into their expression levels in the human microbiome. Direct detection in human samples using sensitive analytical techniques would conclusively prove their production in humans.

The use of sequence similarity networks (SSNs), domain detection via hidden Markov models (HMMs), and protein sequence-structure analysis, allowed the characterization of the enzymes present in BGC8 from *P. gulae* COT-052. As a result, the potential PTM transformations that the precursor peptide could undergo were hypothesized. YcaO from BGC8 was considered to install a backbone amidine between the last two residues of the precursor peptide A8 due to structural similarities with the YcaO ItfB and the shared C-terminal phenylamine of the precursor peptide ItfA (Figure 4; Clark and Seyedsayamdost, 2022). Sequence and structural features (Figure 5; Supplementary Figure S1) also allowed to hypothesize on the role of the rSAM enzyme in installing γ-thioether linkages on the precursor peptide between Cys and Thr residues, as CteB and Tte1186 catalyze in their corresponding precursor peptides (Bruender et al., 2016; Grove et al., 2017; Montalbán-López et al., 2021). Experimental characterization of the reactions catalyzed by these PTM enzymes on the precursor peptides would provide proof of concept for the utility of this bioinformatic approach.

Functional elucidation of the mature products resulting from these precursor peptides will require further investigation. Conducting antimicrobial activity tests against different targets constitutes a first approach to gain insight into their role in antagonistic interactions. It is important to note that, although many experimentally characterized RiPPs display antimicrobial activity (Arnison et al., 2013), alternative functions for RiPPs, such as copper-chelating agents or pheromones, have also been described (Kim et al., 2004; Okada et al., 2005). The presence of suspected signaling proteins in the proximity of the putative precursor peptides in BGC4,5,7 and 8 (Figure 2) could indicate the involvement of the mature peptides on signaling.

In conclusion, this comprehensive *in silico* study has explored the putative RiPP biosynthetic landscape of Bacteroidales bacteria and revealed, upon closer inspection, 9 BGCs with very interesting novel characteristics. These findings provide a template for future experimental investigations in uncovering new biologically active RiPPs from the human microbiota. Furthermore, if they demonstrate antimicrobial activity, these RiPPs offer exciting prospects for translational applications. Considering the increasing evidence linking the microbiota to the onset and progression of different diseases (reviewed in Tremaroli and Bäckhed, 2012; Collins, 2014; Wong and Yu, 2019; Fernandez-Cantos et al., 2021; Read et al., 2021), these RiPPs or their derivatives could be used to selectively modulate the microbiota for therapeutic purposes.

## Data availability statement

The datasets presented in this study can be found in online repositories. The names of the repository/repositories and accession number(s) can be found in the article/Supplementary material.

## Author contributions

MF-C and OK conceived the project. MF-C performed the BAGEL4 screening. MF-C, DG-M, and EG-V selected relevant RiPP BGCs. MF-C and DG-M functionally analyzed the RiPP BGCs. Visualization was carried out by MF-C. YY and LL performed and visualized metagenomic analyses. MF-C and DG-M wrote the original manuscript. OK reviewed the manuscript and supervised the project. All authors contributed to the article and approved the submitted version.

## Funding

This work was supported by Marie Skłodowska–Curie Actions (MSCA) and Innovative Training Networks, H2020-MSCA-ITN-2018 813781 “BestTreat” (MF-C, DG-M, and OK) and by the China Scholarship Council No. 201904910477 (YY).

## Conflict of interest

The authors declare that the research was conducted in the absence of any commercial or financial relationships that could be construed as a potential conflict of interest.

## Publisher’s note

All claims expressed in this article are solely those of the authors and do not necessarily represent those of their affiliated organizations, or those of the publisher, the editors and the reviewers. Any product that may be evaluated in this article, or claim that may be made by its manufacturer, is not guaranteed or endorsed by the publisher.

## Figures and Tables

**Table:** 

AOI	Area of interest
AS	Alignment score
BGC	Biosynthetic gene cluster
ComX	Precursor peptide of the quorum-sensing pheromone
GlyS	S-glycosyltransferase
HMM	Hidden Markov Model
HMP	Human microbiome project
LanB	Lanthipeptide dehydratase
LanC	Lanthipeptide cyclase
LanD	Lanthipeptide C-terminal decarboxylase
LanM	Class II lanthionine synthetase
LasC	Lactam synthetase (involved in Lasso peptide biosynthesis) homolog to Asn synthetase
MSA	Multiple sequence alignment
MFP	Membrane fusion protein
OMP	Outer membrane protein
ORF	Open reading frame
PCAT	Peptidase-containing ATP-binding transporter
PTM	Post-translational modification
RiPP	Ribosomally synthesized and post-translationally modified peptide
RMSD	Root-mean-square deviation
RPKM	Read per Kilobase per Million mapped reads
RRE	RiPP precursor recognition element
rSAM	Radical S-adenosylmethionine
SSN	Sequence similarity network
ThioE	Dehydrogenase (involved in thiopeptide, LAP, and cyanobactin biosynthesis)
TRP	Tetratricopeptide-like helical repeat

## Footnotes

^1^ https://www.beiresources.org/

^2^ http://bagel4.molgenrug.nl/index.php

^3^ https://www.ebi.ac.uk/interpro/

^4^ https://hmpdacc.org/

^5^ https://efi.igb.illinois.edu/efi-est/

## References

[ref1] Alvarez-Sieiro P. Montalbán-López M. Mu D. Kuipers O. P. (2016). Bacteriocins of lactic acid bacteria: extending the family. Appl. Microbiol. Biotechnol. 100, 2939–2951. doi: 10.1007/s00253-016-7343-9 26860942 PMC4786598

[ref2] Arnison G. Bibb M. Bierbaum G. Bowers A. Bugni T. Bulaj G.. (2013). Ribosomally synthesized and post-translationally modified peptide natural products: overview and recommendations for a universal nomenclature. Nat. Prod. Rep. 30, 108–160. doi: 10.1039/C2NP20085F, PMID: 23165928 PMC3954855

[ref3] Arumugam M. Raes J. Pelletier E. Le Paslier D. Yamada T. Mende D. R.. (2011). Enterotypes of the human gut microbiome. Nature 473, 174–180. doi: 10.1038/nature09944, PMID: 21508958 PMC3728647

[ref4] Bartholomae M. Buivydas A. Viel J. H. Montalbán-López M. Kuipers O. P. (2017). Major gene-regulatory mechanisms operating in ribosomally synthesized and post-translationally modified peptide (RiPP) biosynthesis. Mol. Microbiol. 106, 186–206. doi: 10.1111/mmi.13764, PMID: 28787536

[ref5] Begley M. Cotter P. D. Hill C. Ross R. P. (2009). Identification of a novel two-peptide Lantibiotic, Lichenicidin, following rational genome mining for LanM proteins. Appl. Environ. Microbiol. 75, 5451–5460. doi: 10.1128/AEM.00730-09, PMID: 19561184 PMC2737927

[ref6] Belitsky B. R. Sonenshein A. L. (2002). GabR, a member of a novel protein family, regulates the utilization of γ-aminobutyrate in *Bacillus subtilis*. Mol. Microbiol. 45, 569–583. doi: 10.1046/j.1365-2958.2002.03036.x, PMID: 12123465

[ref7] Blin K. Shaw S. Augustijn H. E. Reitz Z. L. Biermann F. Alanjary M.. (2023). antiSMASH 7.0: new and improved predictions for detection, regulation, chemical structures and visualisation. Nucleic Acids Res. 1:gkad344. doi: 10.1093/nar/gkad344, PMID: 37140036 PMC10320115

[ref8] Bobeica S. C. Dong S.-H. Huo L. Mazo N. McLaughlin M. I. Jiménez-Osés G.. (2019). Insights into AMS/PCAT transporters from biochemical and structural characterization of a double Glycine motif protease. Elife 8:e42305. doi: 10.7554/eLife.42305 30638446 PMC6363468

[ref9] Bountra K. Hagelueken G. Choudhury H. G. Corradi V. El Omari K. Wagner A.. (2017). Structural basis for antibacterial peptide self-immunity by the bacterial ABC transporter McjD. EMBO J. 36, 3062–3079. doi: 10.15252/embj.201797278, PMID: 28864543 PMC5641919

[ref10] Bruender N. A. Wilcoxen J. Britt R. D. Bandarian V. (2016). Biochemical and spectroscopic characterization of a radical S-Adenosyl-l-methionine enzyme involved in the formation of a peptide Thioether cross-link. Biochemistry 55, 2122–2134. doi: 10.1021/acs.biochem.6b00145 27007615 PMC4829460

[ref11] Burkhart B. J. Schwalen C. J. Mann G. Naismith J. H. Mitchell D. A. (2017). YcaO-dependent posttranslational amide activation: biosynthesis, structure, and function. Chem. Rev. 117, 5389–5456. doi: 10.1021/acs.chemrev.6b00623, PMID: 28256131 PMC5406272

[ref12] Caetano T. van der Donk W. Mendo S. (2020). Bacteroidetes can be a rich source of novel lanthipeptides: the case study of Pedobacter lusitanus. Microbiol. Res. 235:126441. doi: 10.1016/j.micres.2020.126441, PMID: 32109689

[ref13] Capodagli G. C. Tylor K. M. Kaelber J. T. Petrou V. I. Federle M. J. Neiditch M. B. (2020). Structure–function studies of Rgg binding to pheromones and target promoters reveal a model of transcription factor interplay. Proc. Natl. Acad. Sci. 117, 24494–24502. doi: 10.1073/pnas.2008427117, PMID: 32907945 PMC7533842

[ref14] Chatzidaki-Livanis M. Coyne M. J. Comstock L. E. (2014). An antimicrobial protein of the gut symbiont *Bacteroides fragilis* with a MACPF domain of host immune proteins. Mol. Microbiol. 94, 1361–1374. doi: 10.1111/mmi.12839 25339613 PMC4262677

[ref15] Chatzidaki-Livanis M. Coyne M. J. Roelofs K. G. Gentyala R. R. Caldwell J. M. Comstock L. E. (2017). Gut Symbiont *Bacteroides fragilis* secretes a eukaryotic-like ubiquitin protein that mediates Intraspecies antagonism. MBio 8, e01902–e01917. doi: 10.1128/mBio.01902-17 29184019 PMC5705921

[ref16] Clark K. A. Seyedsayamdost M. R. (2022). Bioinformatic atlas of radical SAM enzyme-modified RiPP natural products reveals an isoleucine–tryptophan crosslink. J. Am. Chem. Soc. 144, 17876–17888. doi: 10.1021/jacs.2c06497 36128669

[ref17] Collins S. M. (2014). A role for the gut microbiota in IBS. Nat. Rev. Gastroenterol. Hepatol. 11, 497–505. doi: 10.1038/nrgastro.2014.40 24751910

[ref18] Cotter P. D. Hill C. Ross R. P. (2005). Bacteriocins: developing innate immunity for food. Nat. Rev. Microbiol. 3, 777–788. doi: 10.1038/nrmicro1273 16205711

[ref19] Coyne M. J. Béchon N. Matano L. M. McEneany V. L. Chatzidaki-Livanis M. Comstock L. E. (2019). A family of anti-Bacteroidales peptide toxins wide-spread in the human gut microbiota. Nat. Commun. 10:3460. doi: 10.1038/s41467-019-11494-1, PMID: 31371723 PMC6671954

[ref20] Donia M. S. Cimermancic P. Schulze C. J. Wieland Brown L. C. Martin J. Mitreva M.. (2014). A systematic analysis of biosynthetic gene clusters in the human microbiome reveals a common family of antibiotics. Cells 158, 1402–1414. doi: 10.1016/j.cell.2014.08.032, PMID: 25215495 PMC4164201

[ref21] Dunbar K. L. Chekan J. R. Cox C. L. Burkhart B. J. Nair S. K. Mitchell D. A. (2014). Discovery of a new ATP-binding motif involved in peptidic azoline biosynthesis. Nat. Chem. Biol. 10, 823–829. doi: 10.1038/nchembio.1608, PMID: 25129028 PMC4167974

[ref22] Edgar R. C. (2004). MUSCLE: multiple sequence alignment with high accuracy and high throughput. Nucleic Acids Res. 32, 1792–1797. doi: 10.1093/nar/gkh340, PMID: 15034147 PMC390337

[ref23] Evans J. C. McEneany V. L. Coyne M. J. Caldwell E. P. Sheahan M. L. Von S. S.. (2022). A proteolytically activated antimicrobial toxin encoded on a mobile plasmid of Bacteroidales induces a protective response. Nat. Commun. 13:4258. doi: 10.1038/s41467-022-31925-w, PMID: 35871068 PMC9308784

[ref24] Fernandez-Cantos M. V. Garcia-Morena D. Iannone V. El-Nezami H. Kolehmainen M. Kuipers O. P. (2021). Role of microbiota and related metabolites in gastrointestinal tract barrier function in NAFLD. Tissue Barriers 9:1879719. doi: 10.1080/21688370.2021.1879719, PMID: 34280073 PMC8489918

[ref25] Gallegos M.-T. Michán C. Ramos J. L. (1993). The XylS/AraC family of regulators. Nucleic Acids Res. 21, 807–810. doi: 10.1093/nar/21.4.807, PMID: 8451183 PMC309210

[ref26] García-Bayona L. Comstock L. E. (2018). Bacterial antagonism in host-associated microbial communities. Science 361:2456. doi: 10.1126/science.aat2456, PMID: 30237322

[ref27] García-López M. Meier-Kolthoff J. P. Tindall B. J. Gronow S. Woyke T. Kyrpides N. C.. (2019). Analysis of 1,000 type-strain genomes improves taxonomic classification of Bacteroidetes. Front. Microbiol. 10:2083. doi: 10.3389/fmicb.2019.02083, PMID: 31608019 PMC6767994

[ref28] Garrido M. C. Herrero M. Kolter R. Moreno F. (1988). The export of the DNA replication inhibitor Microcin B17 provides immunity for the host cell. EMBO J. 7, 1853–1862. doi: 10.1002/j.1460-2075.1988.tb03018.x, PMID: 3049078 PMC457178

[ref29] Gerlt J. A. Bouvier J. T. Davidson D. B. Imker H. J. Sadkhin B. Slater D. R.. (2015). Enzyme function initiative-enzyme similarity tool (EFI-EST): a web tool for generating protein sequence similarity networks. Biochim. Biophys. Acta 1854, 1019–1037. doi: 10.1016/j.bbapap.2015.04.015, PMID: 25900361 PMC4457552

[ref30] Gilbert J. A. Blaser M. J. Caporaso J. G. Jansson J. K. Lynch S. V. Knight R. (2018). Current understanding of the human microbiome. Nat. Med. 24, 392–400. doi: 10.1038/nm.4517, PMID: 29634682 PMC7043356

[ref31] Gilson L. Mahanty H. K. Kolter R. (1990). Genetic analysis of an MDR-like export system: the secretion of colicin V. EMBO J. 9, 3875–3884. doi: 10.1002/j.1460-2075.1990.tb07606.x, PMID: 2249654 PMC552155

[ref32] Grell T. A. J. Goldman P. J. Drennan C. L. (2015). SPASM and twitch domains in S-Adenosylmethionine (SAM) radical enzymes*. J. Biol. Chem. 290, 3964–3971. doi: 10.1074/jbc.R114.581249, PMID: 25477505 PMC4326806

[ref33] Grove T. L. Himes P. M. Hwang S. Yumerefendi H. Bonanno J. B. Kuhlman B.. (2017). Structural insights into Thioether bond formation in the biosynthesis of Sactipeptides. J. Am. Chem. Soc. 139, 11734–11744. doi: 10.1021/jacs.7b01283, PMID: 28704043 PMC6443407

[ref34] Haft D. H. Basu M. K. (2011). Biological systems discovery in Silico: radical S-Adenosylmethionine protein families and their target peptides for posttranslational modification. J. Bacteriol. 193, 2745–2755. doi: 10.1128/JB.00040-11, PMID: 21478363 PMC3133131

[ref35] Havarstein L. S. Diep D. B. Nes I. F. (1995). A family of bacteriocin ABC transporters carry out proteolytic processing of their substrates concomitant with export. Mol. Microbiol. 16, 229–240. doi: 10.1111/j.1365-2958.1995.tb02295.x, PMID: 7565085

[ref36] Hegarty J. W. Guinane C. M. Ross R. P. Hill C. Cotter P. D. (2016). Bacteriocin production: a relatively unharnessed probiotic trait? F1000Res 5:2587. doi: 10.12688/f1000research.9615.1, PMID: 27853525 PMC5089130

[ref37] Heilbronner S. Krismer B. Brötz-Oesterhelt H. Peschel A. (2021). The microbiome-shaping roles of bacteriocins. Nat. Rev. Microbiol. 19, 726–739. doi: 10.1038/s41579-021-00569-w, PMID: 34075213

[ref38] Hols P. Ledesma-García L. Gabant P. Mignolet J. (2019). Mobilization of microbiota commensals and their Bacteriocins for therapeutics. Trends Microbiol. 27, 690–702. doi: 10.1016/j.tim.2019.03.007, PMID: 30987817

[ref39] Jumper J. Evans R. Pritzel A. Green T. Figurnov M. Ronneberger O.. (2021). Highly accurate protein structure prediction with AlphaFold. Nature 596, 583–589. doi: 10.1038/s41586-021-03819-2, PMID: 34265844 PMC8371605

[ref40] Kim H. J. Graham D. W. DiSpirito A. A. Alterman M. A. Galeva N. Larive C. K.. (2004). Methanobactin, a copper-acquisition compound from methane-oxidizing bacteria. Science 305, 1612–1615. doi: 10.1126/science.1098322, PMID: 15361623

[ref41] Kroes I. Lepp P. W. Relman D. A. (1999). Bacterial diversity within the human subgingival crevice. Proc. Natl. Acad. Sci. 96, 14547–14552. doi: 10.1073/pnas.96.25.14547, PMID: 10588742 PMC24473

[ref42] Kuipers O. P. de Ruyter P. G. G. A. Kleerebezem M. de Vos W. M. (1998). Quorum sensing-controlled gene expression in lactic acid bacteria. J Biotechnol Genome Analy Chang Face Biotechnol 64, 15–21. doi: 10.1016/S0168-1656(98)00100-X

[ref43] Li B. Yu J. P. J. Brunzelle J. S. Moll G. N. van der Donk W. A. Nair S. K. (2006). Structure and mechanism of the Lantibiotic Cyclase involved in Nisin biosynthesis. Science 311, 1464–1467. doi: 10.1126/science.1121422 16527981

[ref44] Lubelski J. Rink R. Khusainov R. Moll G. N. Kuipers O. P. (2008). Biosynthesis, immunity, regulation, mode of action and engineering of the model lantibiotic nisin. Cell. Mol. Life Sci. 65, 455–476. doi: 10.1007/s00018-007-7171-2, PMID: 17965835 PMC11131864

[ref45] Lynch S. V. Ng S. C. Shanahan F. Tilg H. (2019). Translating the gut microbiome: ready for the clinic? Nat. Rev. Gastroenterol. Hepatol. 16, 656–661. doi: 10.1038/s41575-019-0204-0, PMID: 31562390

[ref46] Marsh A. J. O’Sullivan O. Ross R. P. Cotter P. D. Hill C. (2010). In silico analysis highlights the frequency and diversity of type 1 lantibiotic gene clusters in genome sequenced bacteria. BMC Genomics 11:679. doi: 10.1186/1471-2164-11-679, PMID: 21118552 PMC3091789

[ref47] McEneany V. L. Coyne M. J. Chatzidaki-Livanis M. Comstock L. E. (2018). Acquisition of MACPF domain-encoding genes is the main contributor to LPS glycan diversity in gut Bacteroides species. ISME J. 12, 2919–2928. doi: 10.1038/s41396-018-0244-4, PMID: 30065309 PMC6246601

[ref48] Mendauletova A. Kostenko A. Lien Y. Latham J. (2022). How a subfamily of radical S-Adenosylmethionine enzymes became a mainstay of Ribosomally synthesized and post-translationally modified peptide discovery. ACS Bio Med Chem 2, 53–59. doi: 10.1021/acsbiomedchemau.1c00045, PMID: 37102180 PMC10114670

[ref49] Meng E. C. Pettersen E. F. Couch G. S. Huang C. C. Ferrin T. E. (2006). Tools for integrated sequence-structure analysis with UCSF chimera. BMC Bioinformatics 7:339. doi: 10.1186/1471-2105-7-339, PMID: 16836757 PMC1570152

[ref50] Merwin N. J. Mousa W. K. Dejong C. A. Skinnider M. A. Cannon M. J. Li H.. (2020). DeepRiPP integrates multiomics data to automate discovery of novel ribosomally synthesized natural products. Proc. Natl. Acad. Sci. 117, 371–380. doi: 10.1073/pnas.1901493116 31871149 PMC6955231

[ref51] Mitchell A. L. Attwood T. K. Babbitt P. C. Blum M. Bork P. Bridge A.. (2019). InterPro in 2019: improving coverage, classification and access to protein sequence annotations. Nucleic Acids Res. 47, D351–D360. doi: 10.1093/nar/gky1100, PMID: 30398656 PMC6323941

[ref52] Montalbán-López M. Scott A. Ramesh S. Rahman R. Heel A. J. Viel H.. (2021). New developments in RiPP discovery, enzymology and engineering. Nat. Prod. Rep. 38, 130–239. doi: 10.1039/D0NP00027B, PMID: 32935693 PMC7864896

[ref53] Mysak J. Podzimek S. Sommerova P. Lyuya-Mi Y. Bartova J. Janatova T.. (2014). *Porphyromonas gingivalis*: major Periodontopathic pathogen overview [WWW document]. J. Immunol. Res. 2014, 1–8. doi: 10.1155/2014/476068, PMID: 24741603 PMC3984870

[ref54] Nakai T. Ito H. Kobayashi K. Takahashi Y. Hori H. Tsubaki M.. (2015). The radical S-Adenosyl-l-methionine enzyme QhpD catalyzes sequential formation of intra-protein sulfur-to-methylene carbon Thioether bonds *. J. Biol. Chem. 290, 11144–11166. doi: 10.1074/jbc.M115.638320, PMID: 25778402 PMC4409272

[ref55] Nicolet Y. Drennan C. L. (2004). AdoMet radical proteins—from structure to evolution—alignment of divergent protein sequences reveals strong secondary structure element conservation. Nucleic Acids Res. 32, 4015–4025. doi: 10.1093/nar/gkh728, PMID: 15289575 PMC506812

[ref56] Oberg N. Precord T. W. Mitchell D. A. Gerlt J. A. (2022). RadicalSAM.org: a resource to interpret sequence-function space and discover new radical SAM enzyme chemistry. ACS Bio Med Chem Au 2, 22–35. doi: 10.1021/acsbiomedchemau.1c00048, PMID: 36119373 PMC9477430

[ref57] Okada M. Sato I. Cho S. J. Iwata H. Nishio T. Dubnau D.. (2005). Structure of the *Bacillus subtilis* quorum-sensing peptide pheromone ComX. Nat. Chem. Biol. 1, 23–24. doi: 10.1038/nchembio709, PMID: 16407988

[ref58] Park Y. J. Kenney G. E. Schachner L. F. Kelleher N. L. Rosenzweig A. C. (2018). Repurposed HisC aminotransferases complete the biosynthesis of some Methanobactins. Biochemistry 57, 3515–3523. doi: 10.1021/acs.biochem.8b00296, PMID: 29694778 PMC6019534

[ref59] Pascal Andreu V. Augustijn H. E. van den Berg K. van der Hooft J. J. J. Fischbach M. A. Medema M. H. (2021). BiG-MAP: an automated pipeline to profile metabolic gene cluster abundance and expression in microbiomes. mSystems 6:e0093721. doi: 10.1128/mSystems.00937-21, PMID: 34581602 PMC8547482

[ref60] Pettersen E. F. Goddard T. D. Huang C. C. Couch G. S. Greenblatt D. M. Meng E. C.. (2004). UCSF chimera—a visualization system for exploratory research and analysis. J. Comput. Chem. 25, 1605–1612. doi: 10.1002/jcc.20084, PMID: 15264254

[ref61] Plat A. Kluskens L. D. Kuipers A. Rink R. Moll G. N. (2011). Requirements of the engineered leader peptide of Nisin for inducing modification, export, and cleavage. Appl. Environ. Microbiol. 77, 604–611. doi: 10.1128/AEM.01503-10, PMID: 21097596 PMC3020565

[ref62] Proctor L. M. Creasy H. H. Fettweis J. M. Lloyd-Price J. Mahurkar A. Zhou W.. (2019). The integrative human microbiome project. Nature 569, 641–648. doi: 10.1038/s41586-019-1238-8, PMID: 31142853 PMC6784865

[ref63] Qin J. Li R. Raes J. Arumugam M. Burgdorf K. S. Manichanh C.. (2010). A human gut microbial gene catalogue established by metagenomic sequencing. Nature 464, 59–65. doi: 10.1038/nature08821 20203603 PMC3779803

[ref64] Read E. Curtis M. A. Neves J. F. (2021). The role of oral bacteria in inflammatory bowel disease. Nat. Rev. Gastroenterol. Hepatol. 18, 731–742. doi: 10.1038/s41575-021-00488-4 34400822

[ref65] Repka L. M. Chekan J. R. Nair S. K. van der Donk W. A. (2017). Mechanistic understanding of Lanthipeptide biosynthetic enzymes. Chem. Rev. 117, 5457–5520. doi: 10.1021/acs.chemrev.6b00591, PMID: 28135077 PMC5408752

[ref66] Roelofs K. G. Coyne M. J. Gentyala R. R. Chatzidaki-Livanis M. Comstock L. E. (2016). Bacteroidales Secreted antimicrobial proteins target surface molecules necessary for gut colonization and mediate competition in vivo. MBio 7:16. doi: 10.1128/mBio.01055-16, PMID: 27555309 PMC4999547

[ref67] Shannon P. Markiel A. Ozier O. Baliga N. S. Wang J. T. Ramage D.. (2003). Cytoscape: a software environment for integrated models of biomolecular interaction networks. Genome Res. 13, 2498–2504. doi: 10.1101/gr.1239303, PMID: 14597658 PMC403769

[ref68] Shisler K. A. Broderick J. B. (2012). Emerging themes in radical SAM chemistry. Current opinion in structural biology, catalysis and regulation •. Proteins 22, 701–710. doi: 10.1016/j.sbi.2012.10.005, PMID: 23141873 PMC4083504

[ref69] Shumaker A. M. Laclare McEneany V. Coyne M. J. Silver P. A. Comstock L. E. (2019). Identification of a fifth antibacterial toxin produced by a single <span class="named-content genus-species" id="named-content-1">*Bacteroides fragilis*</span> strain. J. Bacteriol. 201, e00577–e00518. doi: 10.1128/JB.00577-18 30692177 PMC6436352

[ref70] Sofia H. J. Chen G. Hetzler B. G. Reyes-Spindola J. F. Miller N. E. (2001). Radical SAM, a novel protein superfamily linking unresolved steps in familiar biosynthetic pathways with radical mechanisms: functional characterization using new analysis and information visualization methods. Nucleic Acids Res. 29, 1097–1106. doi: 10.1093/nar/29.5.1097, PMID: 11222759 PMC29726

[ref71] Suau A. Bonnet R. Sutren M. Godon J.-J. Gibson G. R. Collins M. D.. (1999). Direct analysis of genes encoding 16S rRNA from complex communities reveals many novel molecular species within the human gut. Appl. Environ. Microbiol. 65, 4799–4807. doi: 10.1128/AEM.65.11.4799-4807.1999, PMID: 10543789 PMC91647

[ref72] Tamura K. Stecher G. Kumar S. (2021). MEGA11: molecular evolutionary genetics analysis version 11. Mol. Biol. Evol. 38, 3022–3027. doi: 10.1093/molbev/msab120, PMID: 33892491 PMC8233496

[ref73] Teanpaisan R. Narawatthana S. Utarabhand P. (2009). The gene coding for nigrescin produced by *Prevotella nigrescens* ATCC 25261. Lett. Appl. Microbiol. 49, 293–298. doi: 10.1111/j.1472-765X.2009.02657.x, PMID: 19531060

[ref74] Tett A. Pasolli E. Masetti G. Ercolini D. Segata N. (2021). Prevotella diversity, niches and interactions with the human host. Nat. Rev. Microbiol. 19, 585–599. doi: 10.1038/s41579-021-00559-y, PMID: 34050328 PMC11290707

[ref75] Tietz J. I. Schwalen C. J. Patel P. S. Maxson T. Blair P. M. Tai H.-C.. (2017). A new genome-mining tool redefines the lasso peptide biosynthetic landscape. Nat. Chem. Biol. 13, 470–478. doi: 10.1038/nchembio.2319, PMID: 28244986 PMC5391289

[ref76] Tremaroli V. Bäckhed F. (2012). Functional interactions between the gut microbiota and host metabolism. Nature 489, 242–249. doi: 10.1038/nature11552 22972297

[ref77] Turnbaugh P. J. Ley R. E. Hamady M. Fraser-Liggett C. M. Knight R. Gordon J. I. (2007). The human microbiome project. Nature 449, 804–810. doi: 10.1038/nature06244, PMID: 17943116 PMC3709439

[ref78] van Heel A. J. de Jong A. Song C. Viel J. H. Kok J. Kuipers O. P. (2018). BAGEL4: a user-friendly web server to thoroughly mine RiPPs and bacteriocins. Nucleic Acids Res. 46, W278–W281. doi: 10.1093/nar/gky383, PMID: 29788290 PMC6030817

[ref79] van Heel A. J. Montalban-Lopez M. Oliveau Q. Kuipers O. P. (2017). Genome-guided identification of novel head-to-tail cyclized antimicrobial peptides, exemplified by the discovery of pumilarin. Microbial Genomics 3:e000134. doi: 10.1099/mgen.0.000134, PMID: 29177092 PMC5695211

[ref80] Walker M. C. Eslami S. M. Hetrick K. J. Ackenhusen S. E. Mitchell D. A. van der Donk W. A. (2020). Precursor peptide-targeted mining of more than one hundred thousand genomes expands the lanthipeptide natural product family. BMC Genomics 21:387. doi: 10.1186/s12864-020-06785-7, PMID: 32493223 PMC7268733

[ref81] Walsby C. J. Ortillo D. Broderick W. E. Broderick J. B. Hoffman B. M. (2002). An anchoring role for FeS clusters: chelation of the amino acid moiety of S-Adenosylmethionine to the unique Iron site of the [4Fe−4S] cluster of pyruvate Formate-Lyase activating enzyme. J. Am. Chem. Soc. 124, 11270–11271. doi: 10.1021/ja027078v, PMID: 12236732

[ref82] Walsh C. J. Guinane C. M. Hill C. Ross R. P. O’Toole P. W. Cotter P. D. (2015). In silico identification of bacteriocin gene clusters in the gastrointestinal tract, based on the human microbiome Project’s reference genome database. BMC Microbiol. 15:183. doi: 10.1186/s12866-015-0515-4, PMID: 26377179 PMC4573289

[ref83] Waterhouse A. M. Procter J. B. Martin D. M. A. Clamp M. Barton G. J. (2009). Jalview version 2—a multiple sequence alignment editor and analysis workbench. Bioinformatics 25, 1189–1191. doi: 10.1093/bioinformatics/btp033, PMID: 19151095 PMC2672624

[ref84] West A. H. Stock A. M. (2001). Histidine kinases and response regulator proteins in two-component signaling systems. Trends Biochem. Sci. 26, 369–376. doi: 10.1016/S0968-0004(01)01852-7 11406410

[ref85] Wieckowski B. M. Hegemann J. D. Mielcarek A. Boss L. Burghaus O. Marahiel M. A. (2015). The PqqD homologous domain of the radical SAM enzyme ThnB is required for thioether bond formation during thurincin H maturation. FEBS Lett. 589, 1802–1806. doi: 10.1016/j.febslet.2015.05.032, PMID: 26026269

[ref86] Wong S. H. Yu J. (2019). Gut microbiota in colorectal cancer: mechanisms of action and clinical applications. Nat. Rev. Gastroenterol. Hepatol. 16, 690–704. doi: 10.1038/s41575-019-0209-8, PMID: 31554963

[ref87] Wu K.-H. Tai P. C. (2004). Cys32 and His105 are the critical residues for the calcium-dependent cysteine Proteolytic activity of CvaB, an ATP-binding cassette transporter *. J. Biol. Chem. 279, 901–909. doi: 10.1074/jbc.M308296200, PMID: 14570918

