# Supplementary material for: Bioinformatic mining for RiPP biosynthetic gene clusters in Bacteroidales reveals possible new subfamily architectures and novel natural products
Source: Front Microbiol. 2023 Jul 4;14:1219272. doi: 10.3389/fmicb.2023.1219272 (PMC10352776; doi:10.3389/fmicb.2023.1219272)
Supplement: Supplementary file 2 [file Data_Sheet_2.docx]

Supplementary Material


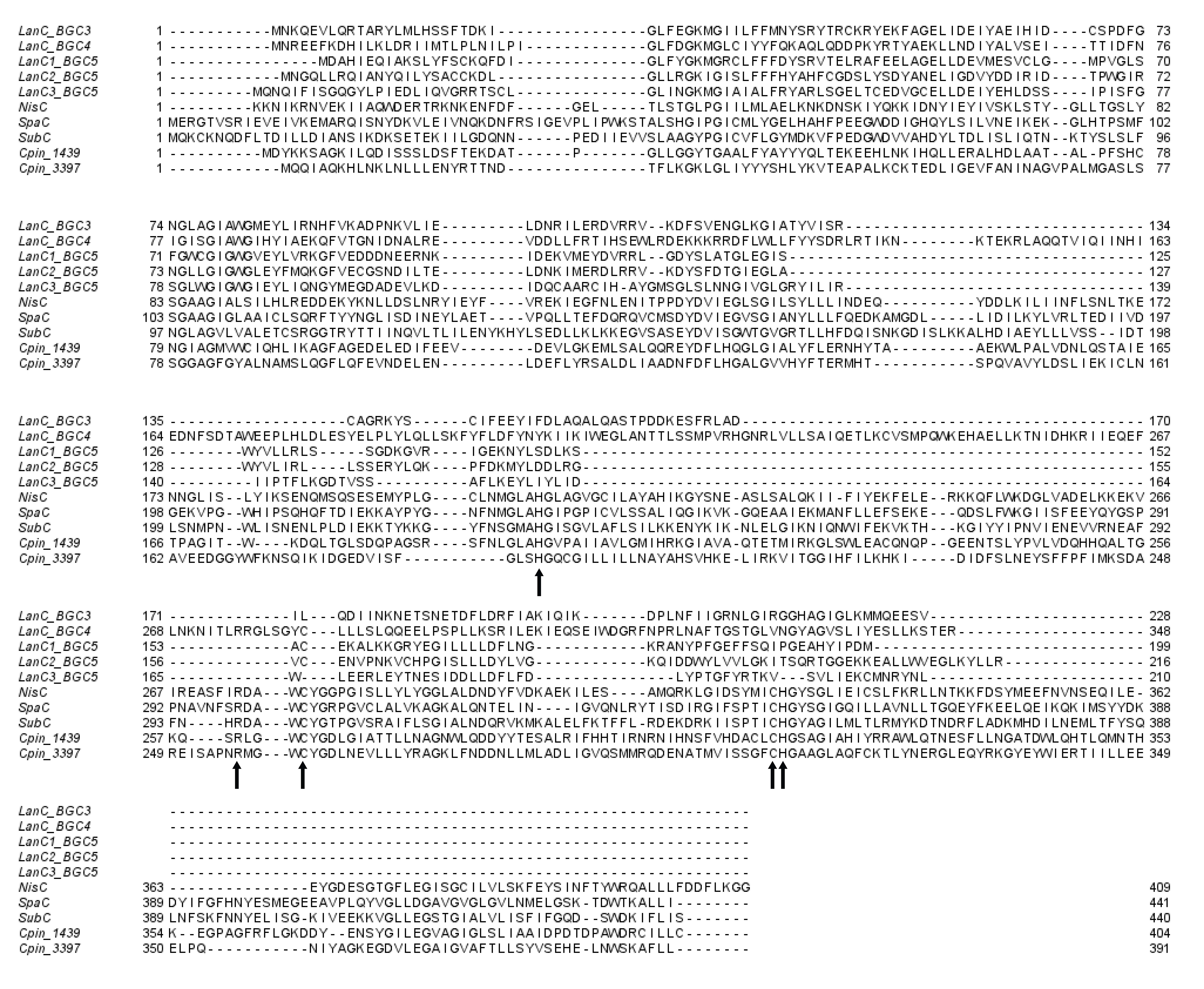


**Supplementary Figure S1.** MSA alignment of LanC enzymes from BGC3,4,5 and LanC experimentally described to be involved in class I lanthipeptide biosynthesis (NisC, SpaC, SubC, Cpin_1439, and Cpin_3397). Relevant residues for LanC catalytic activity are highlighted with arrows.





**Supplementary Figure S2.** (**A**) SSN obtained from radicalSAM.org at an AS of 45. The rSAM from BGC8 is highlighted in red with a size of 100. Experimentally characterized and annotated rSAMs are highlighted in red with a size of 50. (**B**) MSA and Logo of rSAM enzymes from BGC8 and the three closest rSAM annotated (CteB, Tte1186, and QhpD). Residues conserved in at least 3 out of the 4 enzymes are highlighted in red. Cysteine residues suspected to be involved in the coordination of [4Fe-4S] clusters are highlighted in black in the logo.
